# Traditional Retirement or Bridge Employment? Trajectories of Depressive Symptoms After Returning to Work in Korea

**DOI:** 10.1093/geroni/igaf122.1453

**Published:** 2025-12-31

**Authors:** Jeremy Lim-Soh, Hui Xiang Chia, Shannon Ang, Rahul Malhotra, Pildoo Sung

**Affiliations:** Duke-NUS Medical School, Singapore, Singapore; Lee Kuan Yew School of Public Policy, National University of Singapore, Singapore, Singapore; Nanyang Technological University, Singapore, Singapore; Duke-NUS Medical School, Singapore, Singapore; Hanyang University, Seoul, Republic of Korea

## Abstract

Along with an increase in life expectancy and healthy life expectancy among aging populations globally, there is a re-evaluation of the traditional concept of retirement and the value of work in later life. This study explores Korean workers’ exit and re-entry into employment, and their associated trajectories of change in depressive symptoms. Nine waves of the Korean Longitudinal Study of Ageing provided data on the depressive symptoms (Center for Epidemiologic Studies – Depression scale; CES-D) of individuals 45 years and older who left work (mean age after leaving work was 66 years). Group-based trajectory modeling captured heterogeneous changes over time in depressive symptoms, describing the diverse experiences of population sub-groups. We estimated associations between the trajectories and time-invariant individual characteristics and also considered the time-variant role of returning to work. Three trajectory groups were identified: Consistently Low Depressive Symptoms (18%), Consistently Moderate Depressive Symptoms (64%), and Clinically Relevant Depressive Symptoms (19%). The Clinically Relevant Depressive Symptoms group had a CES-D score above the clinical cutoff and were more likely to be self-employed (before retirement), have lower education, and live in non-metropolitan areas. For this group, returning to work had a strong ameliorative effect on depressive symptoms. These findings underscore the dignity of work and the importance of productive aging to mental health. Aging societies should reconsider the overly fixed notion of a universal retirement age and explore policy innovations such as bridge employment and flexible re-employment.

